# Heparan sulfate is a plasma biomarker of acute cellular allograft rejection

**DOI:** 10.1371/journal.pone.0200877

**Published:** 2018-08-07

**Authors:** Andrew S. Barbas, Liwen Lin, MacKenzie McRae, Andrea L. MacDonald, Tracy Truong, Yiping Yang, Todd V. Brennan

**Affiliations:** 1 Department of Surgery, Duke University Medical Center, Durham, NC, United States of America; 2 Department of Medicine, Duke University Medical Center, Durham, NC, United States of America; 3 Department of Surgery, Cedars-Sinai Medical Center, Los Angeles, CA, United States of America; University of Toledo, UNITED STATES

## Abstract

Despite advances in management of immunosuppression, graft rejection remains a significant clinical problem in solid organ transplantation. Non-invasive biomarkers of graft rejection can facilitate earlier diagnosis and treatment of acute rejection. The purpose of this study was to investigate the potential role of heparan sulfate as a novel biomarker for acute cellular rejection. Heparan sulfate is released from the extracellular matrix during T-cell infiltration of graft tissue via the action of the enzyme heparanase. In a murine heart transplant model, serum heparan sulfate is significantly elevated during rejection of cardiac allografts. Moreover, expression of the enzyme heparanase is significantly increased in activated T-cells. In human studies, plasma heparan sulfate is significantly elevated in kidney transplant recipients with biopsy-proven acute cellular rejection compared to healthy controls, recipients with stable graft function, and recipients without acute cellular rejection on biopsy. Taken together, these findings support further investigation of heparan sulfate as a novel biomarker of acute cellular rejection in solid organ transplantation.

## Introduction

Organ transplantation is the definitive treatment for end-stage organ disease. Despite advances in our understanding of immune mechanisms and immunosuppression therapy, graft rejection remains a primary driver of long-term graft failure, with 25–50% grafts lost within 5 years [1, 2]. In the context of ongoing organ shortages, there is a critical need for improved methods for the detection and prevention of graft rejection.

The “gold standard” for diagnosing transplant rejection is the detection of immune injury (e.g. lymphocyte infiltration) on graft biopsy. In kidney transplantation, biopsies are typically performed when serum creatinine (sCr) levels become significantly elevated over baseline. However, sCr is an imperfect indicator of allograft rejection for multiple reasons: it rises late in the process of immune injury, the magnitude of change above baseline can be minimal, and it is not specific for rejection. Protocol biopsies performed at designated intervals can detect rejection prior to elevations in sCr, but are invasive and present additional risk to patients and to the allograft, making this a suboptimal screening approach.

Non-invasive biomarkers of acute allograft rejection are thus necessary to facilitate its early diagnosis and treatment. Although the search for an alternative biomarker has remained elusive, improved understanding of the cellular mechanisms involved in rejection may help inform rational biomarker development. For example, a critical determinant of allograft rejection is the capacity of T cells to extravasate into allografts from the vasculature. Barriers to T cell extravasation into tissues include: 1) the endothelial cell glycocalyx that regulates exposure of adhesion molecules, 2) the subendothelial basement membrane that prevents T cell penetration into tissues, and 3) the extracellular matrix that limits T cell mobility within tissue. A major structural component of these barriers is heparan sulfate (HS), a molecule that is degraded by a specific glycosylase, heparanase (Hpase) [3–6]. Therefore, HS degradation by Hpase is critical for regulating T cell infiltration and graft rejection.

Previously, we have shown that HS is a biomarker of alloimmune injury, mediated by alloreactive T cells during graft-vs-host disease (GvHD) after allogeneic hematopoietic stem cell transplantation (HSCT) [7]. A sentinel diagnostic feature of GvHD is T cell infiltration into tissue, which shares mechanistic similarities to allograft rejection in solid organ transplantation. We found that serum HS levels are elevated in patients with clinical and/or pathological diagnoses of acute GvHD and that HS levels are directly correlated with severity of disease. These findings have led us to hypothesize that HS may also serve as a sensitive and specific biomarker for acute rejection in the context of solid organ transplantation. The purpose of the current study is to investigate the biology of HS and Hpase in allograft rejection, using both a murine cardiac transplant model and investigation of human samples from kidney transplant recipients.

## Materials and methods

### Murine studies

C57BL/6 and BALB/c mice were obtained from the National Cancer Institute at Charles River. All experimental procedures involving the use of mice were done in accordance with protocols approved by the Animal Care and Use Committee of Duke University.

#### Murine heterotopic heart transplantation

An established murine heterotopic heart transplant model was used [8, 9]. Allogeneic transplants were performed by transplantation of donor hearts from BALB/c mice into C57BL/6 recipients (N = 3). Syngeneic transplants were performed using C57BL/6 donor and recipient animals (N = 3). Donor and recipient mice were males between 8 and 12 weeks of age.

#### Donor operation

General anesthesia is induced with isoflurane using a vaporizer. The depth of anesthesia is confirmed by toe pinch. The donor animal is shaved and prepped using Betadine and alcohol. A midline incision is made with the help of surgical scissors. The thorax is opened sharply and the SVC, IVC and the pulmonary veins are localized, dissected from the surrounding tissues, ligated, and divided. The aorta and the main pulmonary artery are transected with appropriate length and flushed with cold saline. The heart is placed in cold saline and used for immediate transplantation.

#### Recipient operation

General anesthesia is induced with isoflurane. The recipient is shaved and prepped using Betadine and alcohol. A midline abdominal incision is made from the symphysis to the pubis. The abdominal aorta and IVC are dissected from the surrounding tissues. The lumbar vessels emerging posteriorly from the great vessels are ligated with 11–0 monofilament suture. An aortotomy is made in the abdominal aorta of the recipient and the donor ascending aorta is anastomosed to the recipient abdominal aorta using 11–0 monofilament suture in an end-to-side fashion. The donor pulmonary artery is anastomosed to the recipient IVC in an end-to-side fashion with running suture of 11–0 nylon. The distal microvascular clamp is removed first followed by the proximal microvascular clamp. Patency and bleeding are inspected, and hemostasis is performed as indicated. The abdominal muscle-fascial layer is closed with a running suture of 5–0 Vicryl. The skin is closed with interrupted suture of 5–0 polypropylene. Topical bupivacaine 0.25% (2 mg/Kg maximum) is placed to the wound margins prior to closure. Buprenorphine SR 0.5–1 mg/kg SC is administered immediately prior to surgery.

#### Method of sacrifice

After experiments were complete, animals were sacrificed by carbon dioxide euthanasia with secondary bilateral thoracotomy.

### Histology

Immunohistochemical (IHC) stains of murine cardiac graft tissue sections were performed on post-operative day (POD) 7 following transplantation. Cryostat sections (10 μM) were dried, fixed in methanol, and incubated for 1 hour in blocking buffer (0.1 M Tris, 0.05 M NaPhos, 0.3% Tween 20, 3% goat serum). Heparanase (Hpase) was detected using polyclonal rabbit anti-Hpase antibody (sc-25825, Santa Cruz Biotechnology, San Diego, CA) and peroxidase-conjugated anti-rabbit IgG (Jackson ImmunoResearch, West Grove, PA). Slides were then developed with diaminobenzidine substrate (SK-4105, Vector Labs, Burlingame, CA) and counterstained with hematoxylin (H-3404, Vector Labs). Quantification of Hpase-expressing graft infiltrating lymphocytes (GILs) was performed by counting the number of cells per high powered field (200X), and comparisons were then made between syngeneic and allogeneic cardiac transplants.

Immunofluorescent staining of cardiac graft tissue was performed using rabbit polyclonal anti-CD3 (ab5690, Abcam, Cambridge, MA) and anti-heparan sulfate (clone F58-10E4, Amsbio, Cambridge, MA), followed by staining with Alexa Fluor 488 conjugated goat anti-rabbit IgG and Alexa Fluor 594 conjugated goat anti-mouse IgM (Thermo Fisher, Waltham, MA). Fluorescent images were obtained using a Zeiss Axio Observer widefield fluorescence microscope and MetaMorph 7 image analysis software (Molecular Devices, Sunnyvale, CA).

### Measurement of serum HS levels

Murine serum levels of HS were determined by heparan sulfate ELISA kits (280564–1, Seikagaku, Tokyo, Japan or SB-E09585h, Cusabio Biotech, Wuhan, China).

### Expression of Hpase by activated CD4 and CD8 T cells

Purified T cells were activated by treatment with 2 μg/mL Concanavalin A (ConA, C5275, Sigma-Aldrich, St.Louis, MO) for 96 hours in RPMI media supplemented with 10% fetal bovine serum.

For Western blots, 15 μg of proteins were resolved by SDS-PAGE and transferred to PVDF membranes (#162–0177, Bio-Rad, Hercules, CA). The antibodies and dilutions used in these experiments were as follows: Rabbit anti-mouse Hpase (1:2000 InSight Biopharmaceuticals Ltd., Rehovot, Israel), HRP-conjugated goat anti-rabbit secondary antibodies (1:10,000 dilution, 111-036-144 Jackson ImmunoResearch, West Grove, PA). Blots were then incubated with SuperSignal West Pico Chemiluminescent Substrate (Thermo Scientific).

For flow cytometry, the following antibodies were used to stain activated CD4 and CD8 T cells: Rabbit anti-mouse Hpase (InSight Biopharmaceuticals Ltd., Rehovot, Israel), donkey anti rabbit-647 (ab150075, Abcam, Cambridge, MA), CD4-BV510 (563106, BD Biosciences, San Jose, CA), CD8-PE (553033, BD Biosciences, San Jose, CA).

### Human studies

De-identified plasma samples from kidney transplant recipients stored in the Department of Surgery Substrate Services Core Biobank under a Duke IRB-approved specimen collection protocol (IRBs #30485 and #71689) were analyzed for this study. Blood samples were obtained at regular time points and also in the setting of a clinically significant event, such as a graft biopsy, infection, or hospitalization. Available clinical data on the subjects include medical history, immunosuppressant medications, hospitalization history, microbiology data, laboratory tests, radiologic findings, and pathology results.

Peripheral blood was also collected from healthy adult donors who signed written informed consented in accordance with the policies of the Duke Institutional Review Board. None of the subjects in this study were from a vulnerable population; all subjects provided written informed consent that was freely given.

Subjects were divided into the following 4 cohorts:

1. Healthy controls (N = 12): Healthy individuals with no history of solid organ transplantation.
2. Stable transplant (N = 24): Kidney transplant recipients with no history of clinically significant events post-transplant and no history of graft biopsy.
3. Rejection (N = 7): Kidney transplant recipients who experienced an episode of acute cellular rejection, proven by graft biopsy using Banff pathologic criteria[10]
4. No rejection (N = 8): Kidney transplant recipients who underwent graft biopsy for elevated sCr and were found to have no evidence of cellular or humoral rejection on final pathologic analysis.

Plasma HS levels were measured for all subjects by ELISA and were compared among all cohorts. For the “Rejection” and “No rejection” cohorts that had undergone graft biopsy, the average and maximum plasma HS and serum Cr levels were compared for a 28 day window (± 14 days) around the date of biopsy. The correlation between plasma HS and serum Cr were subsequently analyzed for the “Rejection” and “No rejection” cohorts.

In a second analysis, serum levels of HS were measured for a cohort of kidney transplant recipients with BK or CMV viremia, to assess whether tissue damage from either of these viral infections impacted blood HS levels. No interventions or changes to the patient’s care plan were made on the basis of results obtained in this study.

### Statistical analysis

For murine studies, comparisons between group means were made using student’s T-test with GraphPad Prism 5.0 (La Jolla, CA). A p-value < 0.05 was considered statistically significant. For human studies, Kruskal-Wallis test was used to test for the overall difference in plasma HS among cohorts. Subsequently, pairwise comparison using Wilcoxon rank sum test was conducted with Bonferroni adjustment for multiple testing. Correlation between plasma HS and serum Cr was calculated using Spearman correlation. Statistical analyses were performed using R Version 3.4.1 software (Vienna, Austria).

## Results

### Murine studies

**Fig 1:**
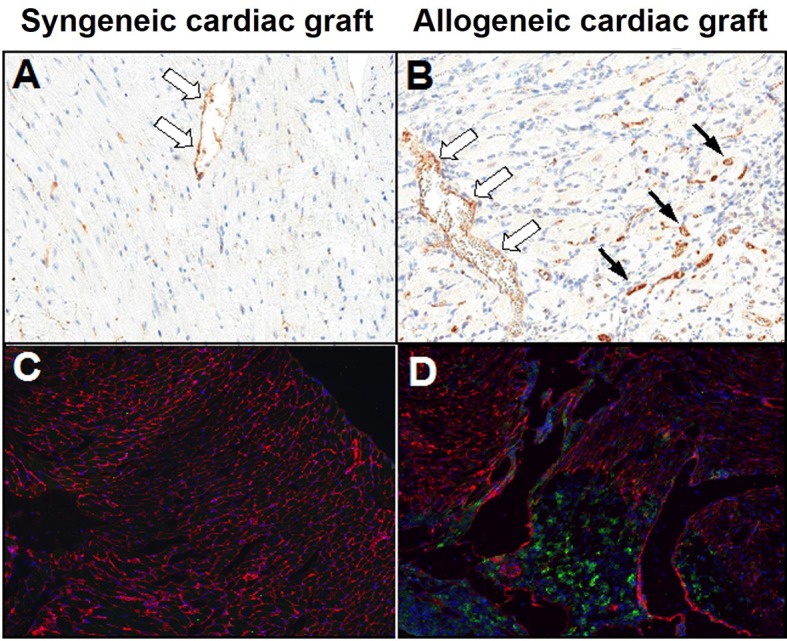
Hpase expression in rejecting murine cardiac allografts versus syngeneic cardiac grafts. Hpase expression is increased in graft-infiltrating lymphocytes (solid arrows) and endothelial cells (hollow arrows) during rejection of allogeneic grafts compared with syngeneic grafts. **A, B)** Hpase staining of syngeneic and allogeneic cardiac transplants on POD 7. Hematoxylin counterstained, 40x mag. **C, D)** HS (Red) present in the graft extracellular matrix is degraded in areas of CD3+ T cell (Green) infiltration during graft rejection compared with control heart. Dapi (Blue) nuclear stain, 40x mag.

We first assessed the expression and activity of Hpase in murine cardiac grafts (allogeneic vs. syngeneic transplants). Immunohistological examination of tissue sections of allogeneic heterotopic heart transplants demonstrates increased levels of Hpase present in rejecting murine cardiac allografts (Fig 1B), compared to syngeneic cardiac grafts (Fig 1A).

**Fig 2:**
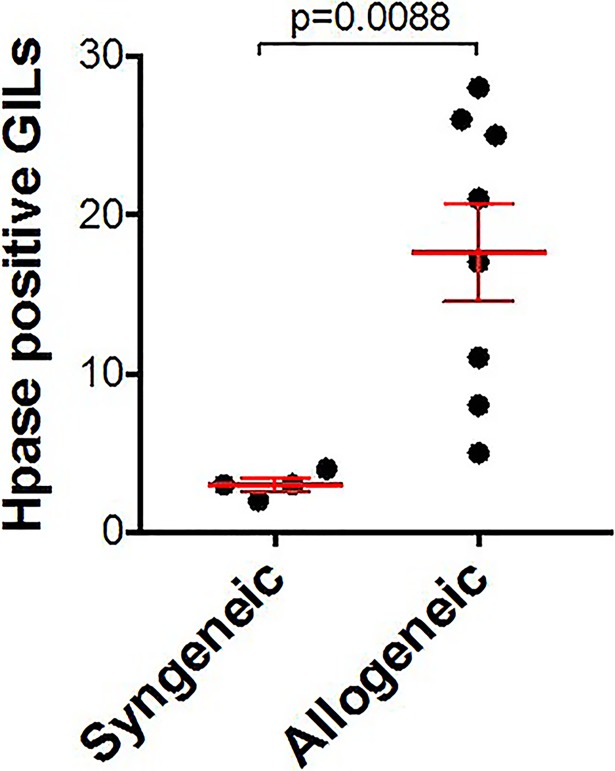
Increased number of Hpase-expressing graft-infiltrating lymphocytes (GILs) in allogeneic versus syngeneic cardiac grafts. Hpase-expressing GILs were counted per high powered field (200X). Mean ± SEM shown in red; comparisons made by unpaired t-test.

While both syngeneic and allogeneic grafts demonstrate Hpase in the endothelium, an increased number of Hpase-expressing graft-infiltrating leukocytes (GILs) was present in rejecting allografts compared to syngeneic grafts (Fig 2).

**Fig 3:**
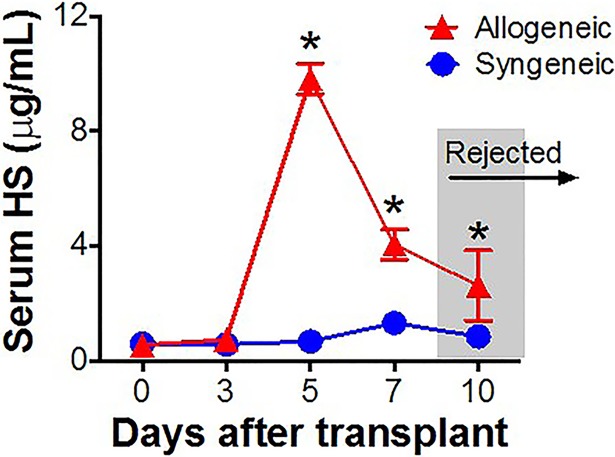
Serum HS levels are elevated in recipients of allogeneic prior to graft rejection. Serum HS levels are elevated following allogeneic (N = 3), but not syngeneic (N = 3) cardiac transplants in mice. (*p<0.05).

We also examined whether HS was degraded in cardiac allograft tissue during T cell infiltration. Detection of CD3+ T cells and HS in rejecting cardiac allograft tissue by immunofluorescence shows dissolution of the abundant intercellular HS by advancing T cell infiltrates (Fig 1C, Fig 1D), providing indirect evidence of robust Hpase activity in these areas. To correlate this increased Hpase activity seen in rejecting allografts with release of HS into the circulation, we measured serum levels of HS in murine cardiac allograft recipients. Serum HS becomes highly elevated during allogeneic, but not syngeneic, cardiac transplantation (Fig 3).

**Fig 4:**
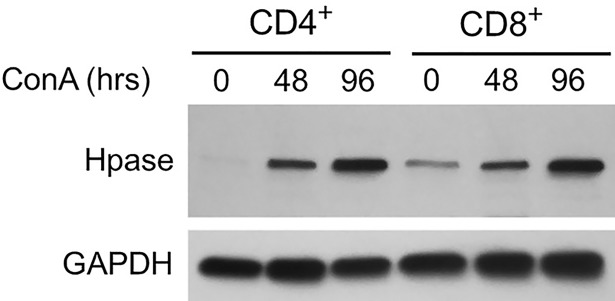
Activated T cell expression of Hpase. Hpase expression is increased in CD4 and CD8 T cells activated by ConA and increases with time. Western blot analysis for Hpase of lysates from purified human CD4+ and CD8+ T cells treated with the pan-T cell activator, ConA, for 0, 48 and 96 hours. Blots were re-probed for GAPDH as a loading control.

**Fig 5:**
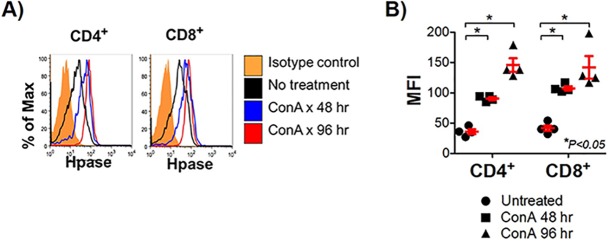
**Cell surface expression of Hpase on activated T cells by flow cytometry.** Cell surface expression of Hpase by activated T cells increases with time. A) FACS analysis of human CD4+ and CD8+ T cells treated with ConA for 0, 48 and 96 hrs for expression of Hpase. B) Mean fluorescent intensity (MFI) of FACS analyses performed in triplicate. (*P<0.05, Unpaired Student’s t-test).

In light of the findings above that suggest degradation of HS is mediated by activated T cells, we examined mouse T cells in vitro to investigate changes in Hpase expression during the activation process. Purified mouse CD4+ and CD8+ T cells were activated using Concanavalin A (ConA), a pan-T cell activator. Both naïve CD4+ and CD8+ T cells demonstrated a robust increase in Hpase expression upon activation (Fig 4, Fig 5).

### Human studies

In order to investigate the potential role of HS as a biomarker of kidney allograft rejection, we measured plasma HS levels in prospectively collected samples from kidney transplant recipients. Plasma HS levels were compared among four cohorts: healthy controls (N = 12), stable post-transplant recipients (N = 24), recipients with biopsy-proven acute cellular rejection (N = 7), and recipients who had undergone biopsy for elevated sCr but without evidence of rejection (N = 8).

**Fig 6:**
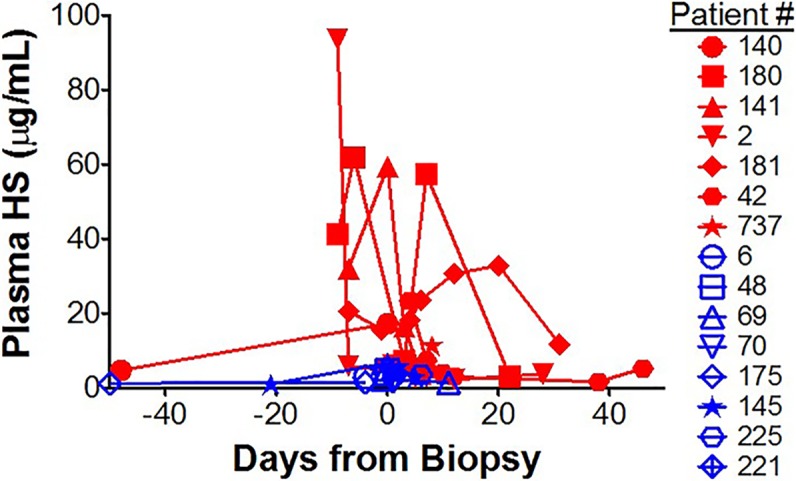
Plasma HS levels in kidney transplant recipients with biopsy-proven acute cellular rejection (red) versus those with no rejection on graft biopsy (blue). Plasma HS levels are elevated in patients with biopsy-proven rejection (red, closed symbols) and not in patients without rejection on biopsy (blue, open symbols). All biopsies were performed for cause (elevated sCr).

We observed significantly elevated plasma HS levels in patients with biopsy-proven acute cellular rejection in comparison to other cohorts (Table 1, Table 2). Of particular interest was the comparison between “Rejection” and “No rejection” cohorts, as all subjects in both cohorts had undergone graft biopsy for an elevation in sCr. As demonstrated in Fig 6, subjects in the “Rejection” cohort demonstrated elevated plasma HS levels at multiple time points, while subjects in the “No rejection” cohort had low plasma HS levels, similar to patients with stable post-transplant function (Fig 6, Table 2).

**Table 1:** Comparison of human plasma HS levels (average and maximum) for the four experimental groups. Kruskal-Wallis test was used to determine if differences existed among groups.

Plasma HS	Healthy control (N = 12)	Stable transplant (N = 24)	Rejection (N = 7)	No rejection (N = 8)	P-value
Average Plasma HS					< 0.0001
Median (IQR)	1.1 (1, 1.4)	1.9 (1.4, 2.5)	21.8 (15.3, 27.3)	2.6 (1.9, 3.8)	
Min, Max	0.5, 2	0.8, 3.7	11.3, 42	1.6, 5.1	
Maximum Plasma HS					< 0.0001
Median (IQR)	1.1 (1, 1.4)	1.9 (1.4, 2.5)	30.7 (20.4, 60.7)	2.7 (2.2, 3.9)	
Min, Max	0.5, 2	0.8, 3.7	11.3, 93.7	1.6, 6.7	

**Table 2:** P-values obtained from pairwise comparisons of plasma HS levels (average and maximum) for four human cohorts using Bonferroni adjustment for multiple testing.

	Healthy control	Stable transplant	No rejection
Average plasma HS			
Stable transplant	0.009		
No rejection	0.004	0.67	
Rejection	0.003	0.0005	0.009
Maximum plasma HS			
Stable transplant	0.009		
No rejection	0.003	0.32	
Rejection	0.003	0.0005	0.009

**Fig 7:**
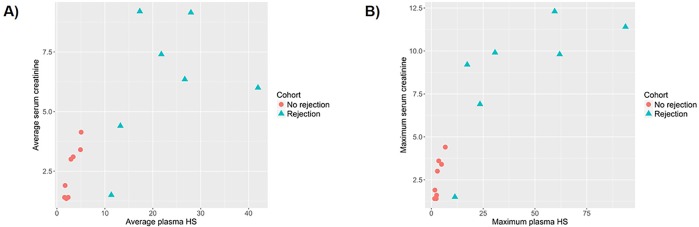
Correlation between plasma HS and serum creatinine. (A) Average plasma HS versus serum creatinine, (B) Maximum plasma HS versus serum creatinine.

When comparing the sCr levels in the “Rejection” and “No rejection” cohorts, we did note a significant elevation in sCr in the “Rejection” cohort, as expected (Table 3). There was a strong correlation observed between plasma HS and serum Cr in “Rejection” and “No rejection” cohorts, with a Spearman correlation of 0.86 and 0.88 for average and maximal values, respectively (Fig 7).

**Table 3:** Comparison of serum Cr (average and maximum) within 14 days from graft biopsy in “Rejection” and “No rejection” cohorts.

Serum creatinine	Rejection (N = 7)	No rejection (N = 8)	P-value
Average serum creatinine			
Median (IQR)	6.3 (5.2, 8.3)	2.5 (1.4, 3.2)	0.009
Min, Max	1.5, 9.2	1.4, 4.1	
Maximum serum creatinine			
Median (IQR)	9.8 (8.1, 10.7)	2.5 (1.6, 3.4)	0.01
Min, Max	1.5, 12.3	1.4, 4.4	

**Fig 8:**
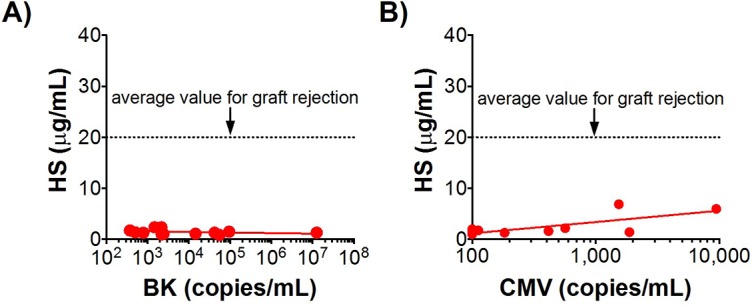
Serum HS is not elevated by BK viral infection (A), and is minimally elevated by CMV infection (B).

In clinical practice, elevations in sCr due to viral infection cannot be distinguished from acute rejection without obtaining a biopsy. In order to establish that HS was specifically associated with acute rejection and not viral infection, we examined HS serum levels in patients with CMV and BK viremia. Both of these viruses are carefully monitored in kidney transplant recipients as they have potential to lead to graft nephropathy. Interestingly, BK viremia did not result in any detectable HS elevation and CMV viremia produced only a mild increase, even at very high titers (Fig 8).

## Discussion

Despite advances in immunosuppressive therapy, 25–50% of solid organ transplants fail within 5 years, primarily due to immune-mediated graft rejection [1, 2]. While current treatment protocols are effective at resolving acute rejection episodes, diagnosis is frequently delayed until advanced and often irreversible graft damage has occurred. Thus, the development of reliable biomarkers to detect transplant rejection prior to overt organ dysfunction is an unmet clinical need with significant potential to enhance the longevity of transplanted organs.

In kidney transplantation, measurement of serum creatinine (sCr) is currently the primary approach in monitoring renal allograft function. However, sCr performs poorly as a biomarker of rejection for several reasons: 1) elevation of sCr occurs late in the process of immunologic injury, 2) the magnitude of change above baseline can be subtle and often less than 2-fold, and 3) elevation of sCr is non-specific for immune injury and can be elevated for other reasons such as dehydration. A biomarker more specific to early immune mediated graft injury would be a significant improvement over current methods for assessing graft rejection. Ideally, such an assay would be measured from a readily available patient sample (e.g. blood), would be reproducible in clinical laboratories, and would be easily interpreted to inform clinical decision making.

In this study, we investigate the potential use of HS as a biomarker of acute cellular rejection. HS is a highly sulfated polysaccharide that is a common component of the extracellular matrix, basement membrane and vascular glycocalyx. Due to the high degree of sulfation, HS carries a substantial negative charge under physiologic conditions and is repulsive to similarly charged cell membranes. Consequently, HS acts as both a physical and an electrostatic barrier to cell migration. Because HS also binds a multitude of proteins, including cytokines, chemokines and growth factors, its degradation affects numerous biological functions, including cell signaling and motility [4, 11–13]. HS released during ECM degradation is also pro-inflammatory through its activation of Toll-like receptor 4 (TLR4), the membrane receptor for bacterial lipopolysaccharide [7, 14–16]. Thus, HS affects multiple aspects of inflammation, including leukocyte recruitment, extravasation and migration towards sites of inflammation [17, 18].

**Fig 9:**
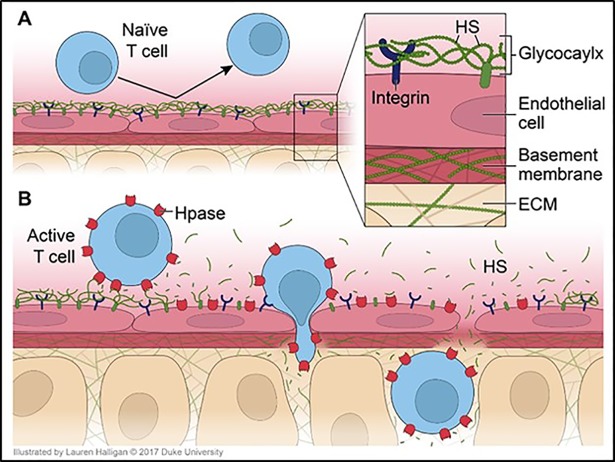
Activated T cells express heparanase (Hpase), facilitating penetration of the glycocalyx and migration into tissues. Heparan sulfate (HS) is released in the process, becoming detectable in serum assays.

In mammals, HS is degraded exclusively by the enzyme heparanase (Hpase), an endo-β-D-glucuronidase [4, 14, 17]. Hpase was originally discovered in activated rat T cells [19], but can be expressed by many other cell types, such as platelets [20], neutrophils [20], dendritic cells [21, 22] and activated endothelial cells [23]. Expression of Hpase by T cells is regulated by antigen recognition. Naïve T cells respond to antigenic activation by de novo synthesis of Hpase, while memory T cells rapidly release Hpase from preformed stores following contact with antigen [24, 25]. Hpase facilitates T cell invasion through tissue barriers [19, 25] and plays a critical role in the pathogenesis of T cell mediated autoimmune disease, such as experimental autoimmune encephalomyelitis [26–28], adjuvant arthritis [27], and type 1 diabetes mellitus [29]. Hpase has also been implicated in the pathogenesis of GVHD following allogeneic bone marrow transplantation, an alloimmune T-cell mediated pathology [30]. Furthermore, Hpase expression on tumor-antigen specific T cells and NK cells has recently been shown to be critical to their ability to invade solid tumors [31, 32]. Thus, there is mounting evidence that Hpase is a critical regulator of tissue invasion by activated T cells. We hypothesize that Hpase activity is necessary for activated T cells to enter tissue by allowing them to penetrate HS rich extracellular barriers, including the vascular glycocalyx, the subendothelial basement membrane and the extracellular matrix (Fig 9). During the process of allograft tissue invasion, HS is released into the circulation by the activity of Hpase and can be detected in the plasma as an early biomarker of rejection.

In this study, we demonstrate that Hpase-mediated release of HS has significant potential as a sensitive and specific biomarker of acute rejection. Our murine cardiac transplant studies demonstrate increased expression of Hpase in rejecting cardiac allografts along with disappearance of tissue HS in areas of T cell infiltration. Accordingly, both naïve CD4+ and CD8+ T cells express Hpase at baseline and demonstrate increased Hpase expression following activation. Furthermore, serum HS becomes highly elevated during allogeneic, but not syngeneic cardiac transplantation, indicating that Hpase activity is specific to the alloresponse and not related to surgical injury or non-specific inflammation.

In human kidney transplant recipients, we similarly found consistent elevation of plasma HS preceding the diagnosis of renal allograft rejection by biopsy. Amongst patients with rejection, plasma HS levels were increased early (>1 week before biopsy) and with a high magnitude relative to baseline (>10-fold higher). In contrast, patients that underwent graft biopsy, but had no histologic evidence of rejection, universally had baseline serum HS levels. Furthermore, BK and CMV viremia did not produce an elevation of HS levels, indicative of the specificity for HS for alloimmune-mediated injury. Most importantly, since all biopsies in this analysis were performed for cause (elevated sCr), these results indicate that had HS been used as the criteria for obtaining a graft biopsy, patients would have been diagnosed and treated earlier for graft rejection.

In conclusion, HS appears to be a sensitive and specific biomarker of allograft rejection. We propose that Hpase-mediated degradation of HS represents a new avenue of investigation in transplantation and holds promise for the development of biomarkers and novel strategies for reducing graft rejection. Specific inhibitors of Hpase may have the potential to lessen T-cell mediated pathology by limiting their access to the extravascular domain.
